# Secondary Spontaneous Pneumothorax Mimicking Lung Herniation

**DOI:** 10.7759/cureus.9141

**Published:** 2020-07-11

**Authors:** Brandon L Walker, Joshua Boster, Azfar S Syed, Nikhil Huprikar, Tyson Sjulin

**Affiliations:** 1 Pulmonary and Critical Care, San Antonio Uniformed Services Health Education Consortium, San Antonio, USA; 2 Internal Medicine, Brooke Army Medical Center, Fort Sam Houston, USA; 3 Internal Medicine, Madigan Army Medical Center, Joint Base Lewis-McCord, USA; 4 Pulmonary and Critical Care, Walter Reed National Military Medical Center, Bethesda, USA; 5 Pulmonary and Critical Care, Brooke Army Medical Center, Fort Sam Houston, USA

**Keywords:** chronic obstructive pulmonary disease, secondary spontaneous pneumothorax, chest tube, talc pleurodesis

## Abstract

Patients with chronic obstructive pulmonary disease (COPD) are at an increased risk for numerous pulmonary complications, including secondary spontaneous pneumothorax (SSP) and lung herniation. We describe the case of a 66-year-old female patient with severe COPD and previous lingula-sparing left upper lobectomy from adenocarcinoma who presented to the emergency department with a painful anterior chest wall mass that varied in size with respiration. This finding, in a patient with a prior history of an invasive thoracic procedure, is suggestive of lung herniation. Further investigation revealed an SSP mimicking the classic physical exam finding of a lung herniation. The patient was deemed a poor surgical candidate; therefore, talc pleurodesis was administered with resolution of the pneumothorax.

## Introduction

A lung herniation is an uncommon phenomenon that results in the outpouching of lung parenchyma through the chest wall, and can be an etiology of chest pain. Typically it is the result of either surgery or trauma, but can rarely occur spontaneously [1]. It presents as a chest wall mass that fluctuates in size with the respiratory cycle. Chronic obstructive pulmonary disease (COPD) has also been associated with lung herniation, and it is theorized that long-term steroid use is a risk factor [2]. Lung herniation most commonly occurs in areas without significant muscle support, such as the eighth and ninth ribs, which lack support from the trapezius and thoracic inlet [3]. Interestingly, no cases of a concomitant secondary spontaneous pneumothorax (SSP) and lung herniation have been reported in literature despite the former occurring with COPD as well.

An SSP is a common complication of COPD and can be the result of forceful coughing in the setting of hyperinflated alveoli. Patients can present with decreased breath sounds on the affected side and may be in respiratory distress. Because these patients have decreased pulmonary function at baseline, this pathology is potentially life threatening, which makes accurate diagnosis paramount [4,5]. We describe an atypical presentation of an SSP mimicking the typical physical exam finding of a lung herniation. 

## Case presentation

**Figure 1:**
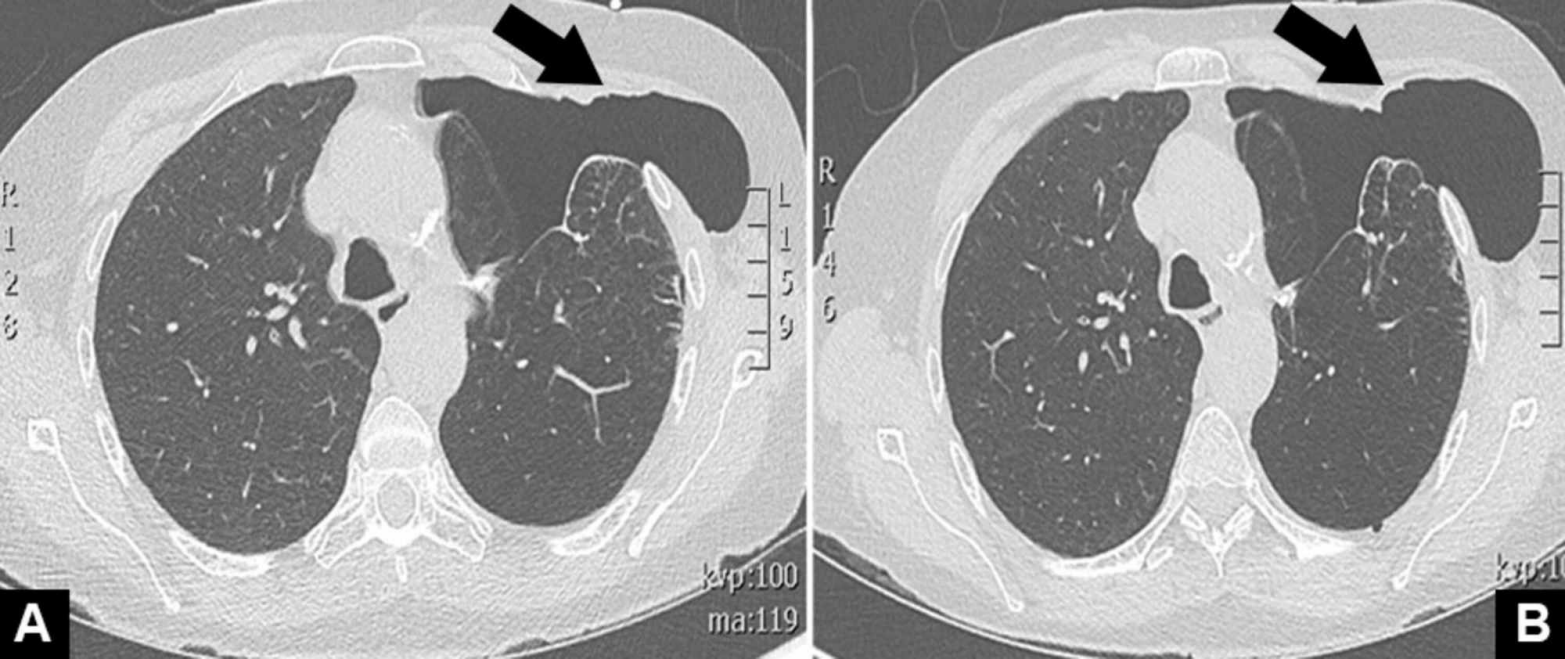
Non-contrast CT scan of the chest during inspiration (A) and exhalation (B) demonstrating left pneumothorax with herniation through the anterior chest wall.

A 66-year-old woman with severe COPD (3D), stage 1A adenocarcinoma of the lung status post minimally invasive lingula-sparing left upper lobectomy one year prior presented for evaluation of a mass on her left lateral chest wall, dyspnea, and pleurisy for the previous three weeks. Her vitals were notable for tachypnea and hypoxia, which required three liters of supplemental oxygen via nasal cannula to maintain an oxygen saturation greater than 88%. Physical examination revealed a softball-sized soft tissue protuberance over the left chest wall in the mid-axillary line. The mass increased in size during exhalation and was reducible on palpation. Pulmonary auscultation was notable for diffuse end-expiratory wheezing and decreased air movement in the left lung field. Complete blood count and complete metabolic panel were unremarkable. Chest radiography revealed a small apical pneumothorax. A subsequent non-contrast chest CT scan demonstrated a left apical pneumothorax with herniation through the chest wall, worsened on exhalation (Figure 1).

**Figure 2:**
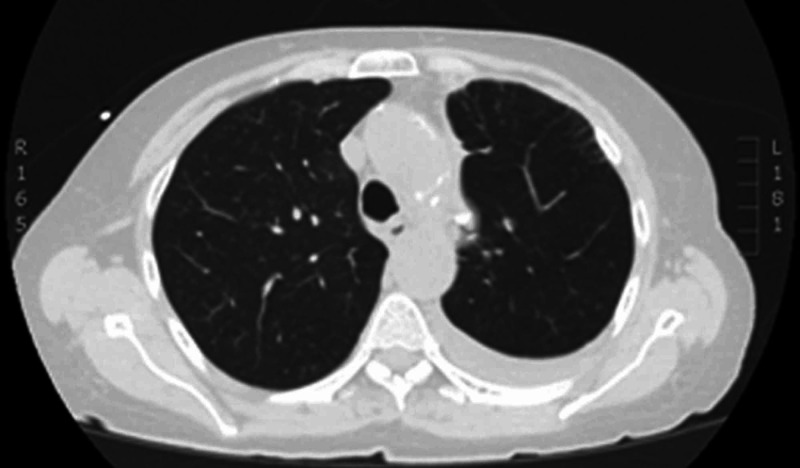
Non-contrast CT scan of the chest status-post talc pleurodesis demonstrating resolution of the secondary spontaneous pneumothorax.

The patient was admitted to the general medicine floor for further intervention. A left-sided anterior apical 14 French chest tube was inserted with resolution of the pneumothorax. Given her comorbidities, the patient was determined to not be a surgical candidate and talc pleurodesis was administered to prevent SSP recurrence. The patient reported improvement in her dyspnea and pleurisy with resolution of the chest wall mass. A non-contrast chest CT was obtained prior to discharge, which revealed resolution of the SSP, and the patient was discharged in a stable condition (Figure 2). 

## Discussion

An SSP is defined as a pneumothorax occurring as a complication of a patient’s underlying lung disease. The list of lung diseases that can lead to an SSP is extensive; however, the most frequent underlying disorders implicated are COPD, cystic fibrosis, pulmonary malignancies, and infectious diseases such as pneumocystis pneumonia. COPD is the underlying disorder in over half of patients presenting with an SSP [6]. It is currently theorized that rupture of apical blebs leads to the development of these pneumothoraces, though alternative explanations have been proposed.

Clinically, patients with an SSP present most often with acute dyspnea and pleuritic chest pain located in the region of the pneumothorax. As these patients have decreased pulmonary reserve at baseline, an SSP tends to present with more severe symptoms than a primary spontaneous pneumothorax. In addition, respiratory failure and hemodynamic instability are not uncommon. 

Lung herniation is a rare event, with less than 300 cases reported within the literature [3]. Although it most often occurs as a result of trauma or as an immediate complication of a surgical procedure, cases of herniation have been documented to occur months to years after thoracotomies. Patients with COPD and chronic cough are at increased risk of developing this complication [3]. It is often asymptomatic but can become quite painful if the herniated tissue becomes incarcerated. The presence of a bulging mass on the chest wall that changes in size with the respiratory cycle is considered to be highly suggestive of lung herniation [7]. Management is plagued by a paucity of evidence, without clear guidelines or evidence-based recommendations for surgical vs. non-operative management [8]. Although reduction of the herniation or resection of the necrotic/incarcerated lung tissue followed by surgical correction of the underlying chest wall defect is the most definitive treatment option, many cases of lung herniation have been successfully managed non-operatively [8].

The management of an SSP differs from that of lung herniation, and therefore definitive diagnosis is required prior to management. Standard treatment of an SSP includes placement of a tube thoracostomy followed by a procedure to prevent recurrence. While open thoracotomy is associated with the lowest risk of recurrence, video-assisted thoracoscopic surgery with bleb stapling and mechanical pleurodesis is the preferred surgical intervention due to the risks associated with open thoracotomy [9]. Medical pleurodesis is an option for patients who are unfit for surgery [10]. If none of these preventive strategies are employed, the recurrence rate of an SSP approaches 50% at three years [11]. In our patient, tube thoracostomy with talc pleurodesis was an effective intervention despite the presence of herniated pneumothorax in the setting of a prior history of minimally invasive lobectomy.

## Conclusions

This report highlights an atypical case of an SSP presenting as an expansible chest wall mass mimicking the classic exam findings associated with lung herniation. When a patient presents with a painful chest wall mass consistent with a lung herniation, it is important to consider SSP in the differential given the differences in treatment strategies. In this patient, tube thoracostomy along with talc pleurodesis resulted in resolution of the pneumothorax and chest wall mass despite the presence of herniated pneumothorax.
